# Awareness of warning signs among suburban Nigerians at high risk for stroke is poor: A cross-sectional study

**DOI:** 10.1186/1471-2377-8-18

**Published:** 2008-05-30

**Authors:** Kolawole W Wahab, Peter O Okokhere, Asuwemhe J Ugheoke, Ojeh Oziegbe, Adedayo F Asalu, Taofeek A Salami

**Affiliations:** 1 Division of Neurology, Department of Medicine, University of Ilorin Teaching Hospital, Ilorin, Nigeria; 2 Department of Medicine, Irrua Specialist Teaching Hospital, Irrua, Nigeria

## Abstract

**Background:**

Although stroke is a leading cause of morbidity and mortality in Nigeria, there is no information on awareness of its warning signs. This study was designed to assess awareness of stroke warning signs in Nigerians at increased risk.

**Methods:**

A hospital-based cross-sectional study conducted at Irrua Specialist Teaching Hospital, in southern Nigeria. Patients with a diagnosis of hypertension, diabetes or both were interviewed for the warning signs of stroke in the outpatient clinic by trained interviewers. The main outcome measure was ability to identify at least one stroke warning sign.

**Results:**

There were 225 respondents with a mean age of 58.0 ± 11.7 years. Only 39.6% could identify at least one stroke warning sign while the commonest sign identified was sudden unilateral limb weakness (24.4%). On multivariate logistic regression analysis, male sex (β = 0.26, 95% CI = 0.14–0.39, p < 0.001) and 11 or more years of education (β = 0.16, 95% CI = 0.03–0.29, p = 0.02) emerged the independent predictors of ability to identify at least one warning sign.

**Conclusion:**

Awareness of stroke warning signs is poor among Nigerians at increased risk for the disease. Efforts should be made to improve on the level of awareness through aggressive health education.

## Background

Stroke is a major cause of morbidity and mortality worldwide especially in developing countries where cardiovascular risk factors are on the increase largely because of adoption of western lifestyle. The WHO estimates that by the year 2030, 80% of all strokes will occur in low and middle income countries [1] which are still battling with the scourge of communicable diseases like HIV/AIDS, malaria and tuberculosis. Although the incidence of stroke is not known in Nigeria, various hospital-based studies have shown that it accounts for as high as 45% of all neurological admissions and 5–17% of medical deaths [2-4] while a case fatality rate as high as 40 percent has been documented [5].

In the management of stroke, time of presentation to the hospital is important as delays often result in poor outcome. The longest phase of delay continues to be the time from symptom recognition to the decision to seek care, and it is in this phase that the most improvement could be achieved [6]. In sharp contrast to the median presentation time of 4.8 hours in a study by Wester et al [7] in Sweden, a median presentation time of 3 days with a range of 1–90 days was reported by Wahab et al in a retrospective study in Nigerians in which the 30-day case fatality rate was found to be 28% [8]. Late presentation to the hospital could be as a result of poor appreciation of stroke warning signs by the victims and relatives. Al Shafaee et al [9] found that 68% of Omani patients at increased risk for stroke could identify at least one warning sign while Pancioli et al [10] found that only 57% of their respondents in a community-based study could identify at least one sign.

In spite of the high case fatality rate from stroke in Nigeria, the authors are not aware of any study that has assessed the level of knowledge of stroke warning signs either in those at high risk for the disease or in the community at large. This study was thus designed to assess the knowledge of warning signs in patients with hypertension, diabetes mellitus or both, conditions that are modifiable risk factors for stroke. Our findings will serve as a basis for health education of those at risk, the general public, the healthcare providers and the policy makers in the country.

## Methods

This study was conducted at the Irrua Specialist Teaching Hospital, Irrua, Edo state in south-south Nigeria. The hospital provides both secondary and tertiary level care to residents of the suburban town of Irrua and the adjoining towns of Ekpoma, Uromi and Auchi. The study protocol was approved by the research and ethics committee of the hospital. From January to March, 2007, consecutively consenting attendees of the medical outpatient unit of the hospital with a diagnosis of hypertension, diabetes mellitus or both were interviewed by the attending medical doctors (which comprised of 6 consultant physicians and an equal number of medical officers). All the interviewers had an initial training on the administration of the study protocol which was followed by a pilot study. Necessary corrections were made after the pilot and it was the final version of the questionnaire that was used in the study. Those who participated in the pilot and those with a past history of stroke were excluded from the study as it was assumed that the former group would have been primed to seek for information on warning signs of stroke while the latter category would have a better knowledge of the warning signs having suffered from the disease before.

Information was obtained in a standardized way with a semi-structured questionnaire. As the south-south part of the country where the study was conducted has many ethnic groups and local languages, the medium of communication was primarily English except in respondents who did not understand English language. In the latter situation, *pidgin *English, which is a common language used in south-south Nigeria even by the uneducated was used. Those who did not understand either English language or *pidgin *English were interviewed in their local language through an interpreter. The interviewers gave no clues to the questions except to give clarifications where necessary. Knowledge of warning signs that have been used in similar studies [9,10] was assessed by the interviewers. These included sudden weakness or numbness of the face, arm, or leg especially on one side of the body, sudden confusion or difficulty in speaking or understanding speech, sudden visual impairment in one or both eyes, sudden difficulty in walking, dizziness, or loss of balance or coordination and sudden severe headache with no known cause. Each respondent was requested to mention as many stroke warning signs as they knew and the correct responses were ticked on the questionnaire by the interviewer. Incorrect signs or inability to mention any sign was reported as "*do not know*". The main outcome measure was ability to identify at least one warning sign.

### Statistics

Data was analyzed with the Statistical Package for the Social Sciences version 11 (SPSS Inc). Frequency tables were generated for the variables of interest. Means and standard deviations were determined. Means were compared using analysis of variance while categorical variables were compared using the Chi-square test. A multivariate logistic regression analysis was done to determine the independent predictor(s) of ability to identify at least one warning sign from among the baseline variables. A p value < 0.05 was taken as a measure of statistical significance.

## Results

### Demographic and clinical characteristics of the respondents

Two hundred and eighty eligible patients were approached for the study but 225 actually participated giving a response rate of 80.1%. The respondents comprised of 96 (42.7%) men and 129 (57.3%) women with an overall mean age of 58.0 ± 11.7 years. The males were significantly older than females (60.3 ± 12.4 vs 56.3 ± 10.8 years, p = 0.01). Thirty five (15.6%) of the respondents were not literate with a significant number of women compared to men being illiterate (21.7% vs 7.3%, p = 0.003). Primary education was the highest educational attainment in 30.2% of the respondents. Overall, 36.0% had at least 11 years of education. Other characteristics of the respondents are as shown in table 1.

**Table 1:** Demographic and clinical characteristics of respondents

**Variable**	**Overall (n = 225)**	**Male (n = 96)**	**Female (n = 129)**	**p value**
Age, years				
Mean ± SD	58.0 ± 11.7	60.3 ± 12.4	56.3 ± 10.8	0.01
≥ 50 years	174 (77.3)	75 (78.1)	99 (76.7)	0.807
Education				
Nil	35 (15.6)	7 (7.3)	28 (21.7)	0.003
Primary	68 (30.2)	25 (26.0)	43 (33.3)	0.239
Secondary	42 (18.7)	26 (27.1)	16 (12.4)	0.005
Tertiary	67 (29.8)	30 (31.3)	37 (28.7)	0.677
Postgraduate	13 (5.8)	8 (8.3)	5 (3.9)	0.156
>11 years	81 (36.0)	38 (39.6)	43 (33.3)	0.334
Family history of stroke	35 (15.6)	18 (18.8)	17 (13.2)	0.254
Current medical diagnosis				
Hypertension	127 (56.4)	54 (56.3)	73 (56.6)	0.960
Diabetes	16 (7.1)	11 (11.5)	5 (3.9)	0.029
Hypertension and diabetes	82 (36.4)	31 (32.3)	51 (39.5)	0.264
Previous health education	63 (28.0)	25 (26.0)	38 (29.5)	0.572

Values are frequency (percentage) except otherwise stated.

### Warning signs identified

As shown in table 2, sudden weakness on one side of the body was identified by 24.4% of the respondents while 13.3% identified dizziness, loss of balance or coordination as a warning sign. No warning sign was identified by 60.4% of the respondents while at least one sign was identified by 39.6%. The other identified warning signs are as presented in table 2.

**Table 2:** Stroke warning signs identified

**Warning sign**	**n (%)**
Sudden weakness on one side	55 (24.4)
Dizziness, loss of balance or coordination	30 (13.3)
Sudden severe headache	27 (12.0)
Confusion and speech difficulty	17 (7.6)
Visual impairment in one or both eyes	5 (2.2)
Do not know	136 (60.4)
No of warning signs identified	
Nil	136 (60.4)
At least 1	89 (39.6)
1	59 (26.2)
2	20 (8.9)
≥ 3	10 (4.4)

### Predictors of ability to identify at least one warning sign

Table 3 shows that on multiple logistic regression analysis, male sex (β = 0.26, 95% CI = 0.14–0.39, p < 0.001) and 11 or more years of education (β = 0.16, 95% CI = 0.03–0.29, p = 0.02) emerged as the independent predictors of ability to identify at least one warning sign.

**Table 3:** Multivariate logistic regression analyses: Predictors of ability to identify at least one warning sign

**Variable**	**β**	**95% CI for β**	**p value**
Age ≥ 50 years	-0.05	-0.213, 0.096	0.45
Male sex	0.26	0.14, 0.39	0.000
≥ 11 years of education	0.16	0.03, 0.29	0.02
Family history of stroke	0.09	-0.05, 0.29	0.16
Health education on stroke	0.09	-0.04, 0.24	0.17
Current medical diagnosis			
Hypertension	0.20	-0.54, 1.30	0.42
Diabetes	0.56	-0.39, 1.50	0.25
Hypertension + Diabetes	-0.42	-1.38, 0.52	0.38

Note: β = beta coefficient, CI = confidence interval

## Discussion

This hospital-based cross-sectional study of Nigerians with potentially modifiable stroke risk factors shows that the knowledge of warning signs of stroke is very poor while male sex and at least 11 years of education emerged as independent predictors of ability to identify at least one warning sign. About 3 out of every 5 respondents could not identify a single stroke warning sign while only 28% reported having ever been educated on stroke by their physicians.

Although the study was in a suburban setting, the literacy rate in the respondents was high and this could be attributed to the possibility that health-seeking behavior is better in the educated thus resulting in a higher number of them seeking treatment at the study centre. However, in spite of this high literacy rate, the overall level of knowledge on ability to recognize the warning signs of stroke is low as only about 40% of the respondents were able to recognize at least one warning sign and a further breakdown showed that 26.2% were able to identify just one sign, two signs were recognized by 8.9% and only 4.4% could identify 3 or more signs.

The observed ability to recognize at least one warning sign of stroke is low when compared to the 68% reported by Al Shafaee et al in a similar study in Oman [9] and contrasts with the findings of Pandian et al [11] who reported that 77% of the respondents in a similar hospital-based survey among patients' relatives in India could identify at least one sign of stroke. The level of knowledge in this study is also low when compared to the 57% reported by Pancioli et al [10] and the 87% reported by Blades et al [12] though these were community-based studies.

In our study, male sex and at least 11 years of education emerged as the independent predictors of ability to identify at least one warning sign. The association with higher level of education is not novel as studies from both developed and developing countries have documented this [9,10,12,13]. However, in contrast to what has been reported from developed countries [10,12], male sex emerged as an independent predictor of better knowledge of stroke warning signs in this study. It is possible that men have a better ability to recognize the warning signs of stroke because of their better education as about twice the number of men compared to women had at least secondary education while about 22% of women did not have any form of formal education. Also, since there is no significant gender difference in the proportion of respondents who had previous health education on stroke, it is likely that men seek other sources of information on the disease in comparison to women. However, since we did not ask for other sources of information on stroke from the respondents, it will be difficult to adduce this reason for the observed difference. Contrary to what is reported from other similar studies [10-12,14], we did not observe any positive relationship between younger age and history of hypertension and the ability to recognize at least one warning sign though we did not assess the influence of the respondents' social class on their ability to identify the warning signs.

A noteworthy observation is the low level of knowledge in spite of the fact that the respondents are already at increased risk for stroke and a high percentage of them are educated. As only 28% of them had previous health education on stroke, it is probable that this low rate of health education and failure of other potential sources of information could have contributed to the observed knowledge gap. Yoon et al reported from a community-based telephone survey in Australia that even among respondents who had a risk factor for stroke, knowledge of stroke warning signs did not differ from that of respondents who had no risk factors [14]. It is also important to note that there is currently no regular public enlightenment programme in any of the news media in Nigeria which is targeted specifically at stroke or its prevention.

Sudden weakness on one side of the body, identified by 24.4%, was the commonest warning sign identified by our respondents and this is comparable to the 26% reported by Kothari et al [15] though the frequency is low when compared to the 65% reported from Oman [9], 60.2% reported from Korea [13] and the 62.2% reported from India [11]. The reason for identification of sudden unilateral weakness by many is probably because it is the most common apparent manifestation of stroke thus many people who have seen a stroke victim with this would be quick to remember it. Only 7.6% of our respondents were able to recognize speech problems as a stroke warning sign in comparison to 90% reported in a study conducted in Ireland [16] though our finding is comparable to the 8% reported from the United States [10] but higher than the 2.4% reported from South Korea [17] and 4.9% reported from India [11]. The reason for these differences is still possibly because of the dominant presentation with weakness on one side of the body which has overshadowed other forms of presentations. Indeed, in some parts of Nigeria, stroke is described simply as a paralysis of the limbs on one side of the body.

The observed poor awareness of warning signs could be the reason why there is a general delay in presenting to the hospital following an acute stroke as has been observed in previous studies in Nigeria [8,18]. Although we are not aware of any study on the influence of duration of symptoms on outcome in acute stroke in Nigerians, we feel that delay in presentation, with the attendant possibility of developing preventable complications could be contributory to the high mortality from stroke in the country at present. This however stems from a poor appreciation of warning signs as Barr et al have shown that those patients who appraise their symptoms as serious tend to have a shorter delay time from symptom onset and hospital admission [19].

The potential limitations of this study are its cross-sectional and hospital-based nature which means that the results might not be completely generalizable to the community. However, because we found poor level of awareness even though more than 80% of our respondents were educated, the level of awareness in the community is even likely to be lower than what we observed meaning that our observations are likely to be valid. A community-based study would be needed to ascertain the generalizability of our results.

## Conclusion

Awareness of warning signs of stroke is poor among Nigerians at high risk while male sex and at least 11 years of education are independent predictors of ability to identify at least one symptom of the disease. Therefore an effort at health education of those at risk and the entire community is advocated so as to improve on the level of awareness and by extension shorten the time between symptom onset and presentation to the hospital in order to reduce the morbidity and mortality from this non-communicable disease. This could be through a joint effort by the physicians and other paramedical personnel attending to high risk patients while community education could be done through regular programmes in the mass media, particularly on radio and television using the appropriate local language to ensure the message is understood even by the uneducated. The government, policy makers and professional associations in the country could greatly assist in the latter regard.

## Competing interests

The authors declare that they have no competing interests.

## Authors' contributions

KWW conceived and coordinated the study, analysed the data and drafted the initial manuscript. All authors were involved in initial literature search and collection of data. Review of initial manuscript for major intellectual content was done by POO, AJU, OO, AFA and TAS. All authors read and approved the final manuscript.

## Pre-publication history

The pre-publication history for this paper can be accessed here:


